# Food Access Patterns and Barriers among Midlife and Older Adults with Mobility Disabilities

**DOI:** 10.1155/2012/231489

**Published:** 2012-09-26

**Authors:** Deborah L. Huang, Dori E. Rosenberg, Shannon D. Simonovich, Basia Belza

**Affiliations:** ^1^Veterans Affairs Puget Sound Health Care System, Geriatric Research, Education and Clinical Center (S-182), 1660 South Columbian Way, Seattle, WA 98108, USA; ^2^Group Health Research Institute, 1730 Minor Avenue, Suite 1600, Seattle, WA 98101, USA; ^3^Department of Biobehavioral Nursing and Health Systems, School of Nursing, University of Washington, P.O. Box 357266, Seattle, WA 98195, USA; ^4^Health Promotion Research Center, University of Washington, P. O. Box 357266, Seattle, WA 98195, USA

## Abstract

We examined where midlife and older adults with a mobility disability accessed food outside the home in King County, Washington, USA, how they travelled to these food destinations, and facilitators and barriers to food access using qualitative interviews. Thirty-five adults aged ≥50 years with a mobility disability (defined as use of an assistive device for mobility) were interviewed. Supplemental objective information was obtained from a Global Positioning System device worn by participants for 3 days. Participants primarily accessed food at grocery stores, restaurants, and coffee shops/cafés. The most common transportation modes were walking, obtaining a ride from friends, motorized chair/scooter, and public transit. Location and proximity of food destinations were factors affecting participants' ability to access these destinations. Adequate space, ease of entry, available amenities such as restrooms, and helpful people were facilitators for participants to access food outside the home.

## 1. Introduction

The ability to access food is essential to life and health. Food access is defined as “having sufficient resources to obtain appropriate foods for a nutritious diet” and is one dimension of food security (defined as “including both physical and economic access to food that meets people's dietary needs as well as their food preferences”) [1]. The prevalence of food insecurity was 7.9% among U.S. households with elderly individuals in 2010 [2]. Difficulty accessing food may negatively affect the nutritional status and health outcomes [3], especially in older adults [4, 5]. Food insecurity was previously reported to be associated with functional impairments in U.S. older adults [6]. Accessing food is a complex process requiring a location to access food, adequate financial and transportation resources, and the cognitive ability to plan and carry out accessing food.

 Older adults and adults with a disability may have more difficulty accessing food due to physical limitations [7], inability to drive, financial limitations, and environmental limitations, among other factors. Physical limitations are more common in older age: in 2009, 38.4% of adults ≥65 years reported physical limitations (difficulty in stooping, lifting, reaching, grasping, or walking, but no limitations in carrying out activities of daily living or instrumental activities of daily living) [8], and 60.8% of community-dwelling adults ≥65 years in 2010 reported difficulty with at least one basic action (defined as movement, emotional, sensory [seeing or hearing], or cognitive) [9]. Lower income is also more common in older age and in adults with disability [10]. As these physical and financial limitations increase, environmental limitations may be more difficult to overcome.

Available transportation is another important factor for food access, especially considering that reliance on cars is high in the U.S. [7, 11, 12]; adults may be less likely to drive with increasing age due to disability and cost. An Australian study reported that lack of car access was a barrier to accessing food, and that older adults were more likely to lack car access [11]. Another study of 16 Australian adults reported that they most commonly walked to food shops, but also used several other transportation modes [12]. The built environment (human-made structures, e.g., buildings, roads, etc.) of potential food access destinations may also impair the ability of older adults and adults with mobility disabilities to access food. One study of grocery and convenience stores in Chicago found that while 63% of stores had an accessible entrance, none of the stores met all accessibility criteria [13]. While transportation and accessibility are key concepts in food access research, the perceptions of these characteristics are not well described. To date, there are few studies that explore how the built environment impacts the ability of adults with a mobility disability to access food.

We hypothesize that midlife and older adults with a mobility disability may face difficulty in accessing food. Food access was defined as going outside the home to obtain or consume food. The purposes of this study were to (1) increase understanding of where midlife and older adults with a mobility disability access food; (2) how they travel to food access destinations; (3) facilitators and barriers (including built environment) to accessing food. We undertook a qualitative study using interviews of adults aged ≥50 years with a mobility disability (defined as requiring an assistive device for mobility). Qualitative methods were used due to the lack of available studies about how this population perceives the built environment and to obtain robust descriptions of facilitators and barriers to accessing food. This approach is not frequently used in studying food access.

## 2. Methods

The Built Environment, Accessibility, and Mobility Study (BEAMS) was a pilot study conducted from October 2010 through September 2011 in King County, Washington, USA. Study procedures were approved by the Institutional Review Board at the University of Washington. The primary goal of BEAMS was to better understand built environment facilitators and barriers to physical activity in midlife and older adults with mobility disability by obtaining their perspectives [14]. Two sets of questions were also asked about utilitarian activities for goods and services, including food destinations. We conducted a qualitative study utilizing in-depth individual interviews in addition to obtaining participant travel information from Global Positioning System (GPS) devices. We chose interviews as the best method to obtain detailed information from study participants about their perceptions of the built environment, since there are few published studies about this topic.

 Study eligibility criteria were as follows: age ≥50 years, use an assistive device for mobility, leave home ≥3 days/week, reside in King County, Washington, speak and read English, and allow study researchers to visit their residence. Our target study enrollment was 25–40 participants with the goal of reaching theme saturation (i.e., no additional new themes or concepts are generated from additional participant interviews) [15]. Participants were purposefully recruited through study announcements in relevant organizational e-newsletters (e.g., senior center newsletters, Arthritis Foundation), as well as flyers distributed at senior centers, community events and other locations where older adults meet. Additionally, we recruited participants with a range of disability types, who used different assistive devices, and resided in diverse types of neighborhoods (e.g., walkability, income) in order to obtain a variety of perspectives.

 Individuals who were interested in the study, met eligibility criteria, and gave verbal consent to participate were mailed a written consent form, GPS device (Qstarz BT-Q1000XT, Qstarz International Co., Ltd., Taipei, Taiwan), GPS instructions for use, and a prepaid return envelope. Participants wore a GPS device for 3 days (2 weekdays and 1 weekend day) prior to their interview to acquire objective information about where participants travelled to destinations. The home interview was conducted after the signed consent form and GPS device were returned by mail. Prior to the interview, study researchers printed color maps from the GPS device to serve as interview prompts.

 Semistructured interviews were conducted at participant homes by two study researchers. One researcher interviewed the participant, while the other researcher took detailed notes using a laptop computer. The interviewer was in the participant's direct sight line, while the researcher taking notes sat to the side. Interviewers had a Bachelor's degree or higher in a health-related field (e.g., nursing, clinical psychology) and underwent a minimum of 5 hours of training, which included practicing with the interview protocol, conducting a formal practice interview with observation and feedback from the principal investigators (D. E. Rosenberg, B. Belza), and observing one of the primary investigators conduct an interview. The note takers were trained to use an Excel template in which they could quickly and easily fill in responses to each item of the interview guide. This training included being observed and provided with feedback during at least one-structured-practice interview session.

The interview protocol consisted of open-ended questions about facilitators and barriers to (1) accessing and using destinations while using the GPS device (up to 3 locations were discussed); (2) accessing utilitarian locations (e.g., grocery stores, shops) in the neighborhood (up to 10 locations discussed); (3) use of indoor and outdoor physical activity locations in the neighborhood (interview protocol available upon request from D.R.). In addition to assessing barriers and facilitators, we asked participants what transportation mode they used to travel to each location visited while wearing the GPS device. Immediately upon completion of the interview, the two study researchers who conducted the home visit debriefed about the interview and each confirmed the interview notes' content and accuracy.

 Additional participant demographic and background data collected with a self-reported, written survey were age, race/ethnicity, checklist of health conditions, and checklist of assistive devices used. Census 2000 data were used to determine median household income at the census tract level. (Census tracts are small statistical subdivisions of counties with 2,500–8,000 persons, which are “designed to be homogeneous with respect to population characteristics, economic status, and living conditions.” [16]). Census 2000 data were the most current census data available during the study period. Neighborhood walkability scores were generated using walkscore.com. Walkability is a measure of how easy it is to walk and live in an area [17]. Walkscore may be used to estimate proximity to walkable destinations [18]; scores range from 0 to 100 with higher scores signifying greater walkability. The U.S. Department of Agriculture's Food Desert Locator [19] was used to determine whether any study participants lived in food deserts. Food deserts are defined as “a low-income census tract [poverty rate ≥20% or median family income ≤80% of area's median family income] where a substantial number or share of residents has low access to a supermarket or large grocery store [≥500 people and/or ≥33% of census tract's population resides >1 mile from a supermarket or large grocery store]” [19].

For the purpose of this study, typed interview notes were specifically analyzed pertaining to accessing food outside the home. Content analysis (i.e., analyzing themes and concepts) was used to develop a start list of codes [20] using an inductive reasoning approach after review of the interview notes. Codes pertained to participants' reports of where they accessed food outside their home, how they travelled to food access destinations, their reasons for visiting food access destinations, and their perceptions of the indoor built environment at food access destinations. These codes were subcategorized into facilitators and barriers to accessing food destinations. Two study researchers (D. L. Huang, D. E. Rosenberg) reviewed the start list of codes and achieved consensus. As new themes were introduced by participants, codes were further refined during coding using both inductive and deductive reasoning approaches. An audit trail tracking the decision-making process was kept throughout the coding process. Coding was performed by two study team members (D. L. Huang, D. E. Rosenberg) and results were discussed to determine consensus for final coding. We achieved theme saturation as well as interrater agreement in coding. Descriptive analyses of demographic data and assistive device use were performed using SPSS version 19 (IBM, Armonk, NY, USA).

## 3. Results

Thirty-five participants were interviewed. Characteristics of study participants are shown in Table 1. Participants had a mean age of 66.8 years, were predominantly female and Caucasian, and used a variety of mobility assistive devices (some participants used more than one assistive device). The majority of participants reported not driving. Two participants lived in food deserts [19], and four participants lived in retirement facilities that provided food on-site in a dining room. These facilities were not counted as an out-of-home location or place to obtain food for home consumption.

### 3.1. Types of Food Access Locations

**Figure 1:**
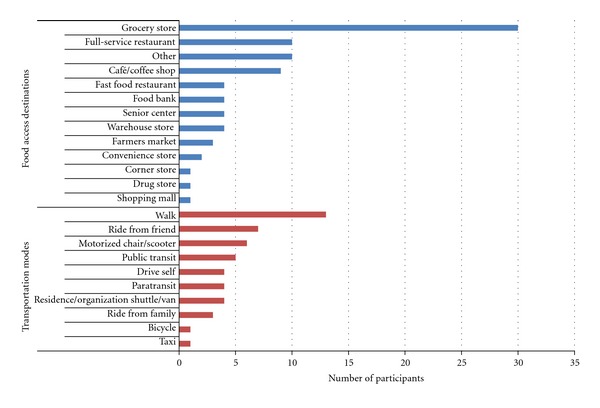
Counts of food access destinations and transportation modes used by study participants.

Participants accessed a variety of locations to obtain food (Figure 1). Grocery stores were the most commonly accessed out-of-home food location by study participants. Several participants reported accessing 2 or more different grocery stores. Participants also visited food banks, warehouse stores, farmers markets, convenience stores, corner stores, and drugstores to obtain food (see Figure 1 for the number of participants who reported going to various types of food locations). Destinations accessed for food consumed outside the home included restaurants (both full-service and fast food), cafés/coffee shops, senior centers, and shopping malls. Most participants went to more than one type of destination to access food. Participants who lived in facilities that provided on-site meals also accessed food outside the home at locations such as grocery stores and restaurants. Three participants reported that family members regularly brought food to their homes (e.g., family purchased groceries for participant).

### 3.2. Types of Transportation to Food Access Destinations

Transportation used by participants included active (e.g., walking) and passive (e.g., using a motorized chair or scooter, paratransit) modes to access food locations (Figure 1). The majority of participants reported using one transportation mode, but others reported using 2 or more modes of transportation to access food. These participants used combinations of walking, public transit, paratransit, rides from friends or family, and vans or shuttle services provided either by their residence or an organization (e.g., senior center).

### 3.3. Factors Impacting Choice of Food Destinations

Reasons for visiting (or not visiting) food access destinations were categorized into three major themes: destination factors, participant factors, and outdoor built environment (Table 2). Barriers to accessing food destinations included all three themes; facilitators to access included destination and participant factors.

#### 3.3.1. Destination Factors

Location and proximity were frequent destination factor subthemes. Some participants reported visiting food destinations because of their proximity to other locations with goods and services, such as drugstores and banks. Cost was another common subtheme of destination factors: participants chose where they shopped for groceries and ate meals outside the home (e.g., restaurants, senior centers) based on cost.

#### 3.3.2. Participant Factors

Subthemes included participants' preference for certain stores, usually based on product availability and selection. Some participants also reported going to food access destinations in order to socialize and stay active.

#### 3.3.3. Outdoor Built Environment

Outdoor built environment characteristics were primarily perceived as (1) barriers to accessing certain food destinations and (2) transportation barriers to food destinations. These included lack of sidewalks, obstructed roads (e.g., parked vehicles, construction), hills and lack of public transit to food destinations.

### 3.4. Facilitators and Barriers of Food Access Destinations

Four themes emerged as key facilitators and barriers for visiting food access locations (Table 3). These included (1) space, (2) entry/accessibility, (3) amenities, (4) people.

#### 3.4.1. Theme 1: Space

This theme included participants' general perceptions of (a) adequacy of space to carry out their intended activity (e.g., grocery shopping, drinking coffee at a coffee shop) while using a mobility assistive device, (b) ease or difficulty navigating the destination, and (c) general destination features. Participants reported that wide, unobstructed aisles facilitated shopping in grocery stores, while a common barrier was narrow aisles. Obstructed aisles, including displays in aisles and other shoppers' carts, were also commonly reported barriers to navigating grocery stores. Other examples of barriers included crowds and closely spaced tables and chairs at restaurants. Additional facilitators to navigating food destinations included elevators to access different floors, no stairs (destination on a single level), and stable product layouts. Built environment barriers to navigating destinations included escalators or stairs to access different floors, small elevators, changes in product layouts (e.g., items periodically moved to different locations in store), a large number of store aisles, and poor signage. General features that participants reported to be facilitators included clean flooring (slippery or uneven flooring were barriers), adequate light, and whether the destination followed the American with Disabilities Act Standards for Accessible Design.

#### 3.4.2. Theme 2: Entry/Accessibility

Ease of entry to the food destination was a common theme among participants. Doors and entries were the two subthemes. Facilitators were automatic doors, lightweight doors, entries that were conveniently located, flat, at street level, and without an entry ramp or stairs. Barriers were heavy doors, doors that opened outward, two sets of entry doors, raised thresholds and door mats, poorly located entries, and the presence of stairs. One participant reported that an entry ramp was a barrier, because it was difficult to simultaneously open the entry door and control her assistive device on the ramp.

#### 3.4.3. Theme 3: Amenities

A number of participants reported using shopping carts as an assistive device for support and mobility in stores. Participants also stated that the availability of electric shopping carts was a facilitator to food shopping and the lack of these carts was a barrier. Participants reported availability of seating and accessible restrooms as facilitators for accessing and utilizing food destinations. Lack of available seating to rest was a barrier. Seat height (which affects the ability to sit down and rise from seating) and space to sit with an assistive device were subthemes. Accessible restrooms with adequate space and doors that were easy to open were facilitators for use. Restrooms without accessibility features were reported as barriers to use. One participant specifically mentioned poor placement of grab bars affected his ability to transfer to and from the toilet, as well as urinals not designed for those using wheelchairs. An additional amenity facilitator was the availability of accessible drinking fountains.

#### 3.4.4. Theme 4: People

The major theme was quality of service given by food access destination staff/employees. Poor or suboptimal service was reported by a few participants, though typically not at grocery stores: one participant reported rude volunteers at a food bank. Examples of suboptimal service included long waiting times for service and unavailability of table service. One participant mentioned avoiding certain food destinations because table service was not available; she preferred going to destinations with table service due to difficulty carrying food to a table while using an assistive device. Good service was a facilitator reported by several participants, which included destination staff/employees, and paratransit and shuttle service drivers. Examples of good service included assistance with bringing groceries to the participant's vehicle, reaching items on high shelves, and opening doors. Some participants reported visiting certain grocery stores because employees knew them. Another theme noted was having helpful family, friends, or caregivers to access food destinations (facilitator); inattentive fellow customers were a barrier. One participant mentioned nearly being knocked over by another customer's shopping cart.

## 4. Discussion

Midlife and older adults with mobility disability in our study accessed a variety of food destinations and utilized several different modes of transportation to these destinations. Many participants accessed more than one type of food destination and used more than one transportation mode. This included participants who lived in facilities that provided on-site meals; these participants also obtained groceries from facility “field trips” to grocery stores or from family members who brought groceries to their home. Additionally, many of those who accessed grocery stores reported going to more than one store. This demonstrates how our study participants adapted to carrying out these instrumental activities of daily living (shopping and transportation), despite their mobility and transportation limitations. While most participants used passive forms of transport (e.g., by automobile) to food destinations, thirteen participants reported walking to food destinations. This indicates that having food locations in proximity to where older adults live can promote physical activity as well as food access among people with mobility disabilities. An interesting concept that was not fully captured in these interviews was the amount of time some participants spent accessing food. For example, some participants reported devoting a certain day of the week to obtain food; one participant reported regularly visiting more than one food bank.

 Location and proximity of food access destinations were important factors for our study participants. Interestingly, some participants reported accessing food in combination with carrying out other utilitarian activities such as going to the bank or to the doctor. One participant reported that she preferred to visit the grocery stores near a senior center (not in her neighborhood), because it was close to several other locations with goods and services. The two participants who lived in food deserts also spoke about the importance of location and proximity of food access destinations. Cost was another important factor for study participants in determining where they chose to access food. This is not surprising given that older adults are more likely to have a fixed income. Some participants also reported travelling farther from their residence to access food at a lower-cost destination. Preference for certain food access destinations was also important, but location, proximity, and cost seemed to carry greater weight in where our study participants chose to access food.

 The perceived indoor built environment barriers and facilitators pertained to adequate space, ease of accessing multiple levels at destinations, ease of entry, and available amenities at food access destinations. Door features were frequently noted by our study participants, particularly doors that were difficult to open due to heavy weight and location (e.g., located at top of entry ramp). Amenities such as available seating and accessible restrooms were also important to participants. An interesting nonbuilt environment finding was the impact of helpful people on accessing food destinations. Participants appreciated friendly employees at these destinations and availability of extra assistance. Additionally, participants liked the extra help they received from paratransit and shuttle/van drivers. For example, one participant reported that the van driver for a senior center carried her groceries from the store to the van and from the van to her home.

Our findings must be couched within the context of the study limitations. A limitation of our study was that we did not audio record and transcribe the interviews due to participant concerns. Early in the recruitment process, our target population voiced concerns about being audio recorded. Many participants received government assistance for housing and transportation, and were concerned about the potential implications of being audio recorded for receiving these benefits. However, the credibility of our findings was enhanced by our training procedures for both interviewing and taking interview notes. In addition, the coders achieved consensus during the coding process even with their differing levels of interview involvement (D.H. was not involved in participant interviews, D. E. Rosenberg either conducted or took notes for approximately one-third of the interviews) and their different disciplinary backgrounds (geriatric medicine, clinical psychology, and public health).

An additional limitation of this study was that BEAMS' primary focus was to understand the impact of built environment on physical activity, though participants were interviewed about utilitarian activities (including food access). This likely influenced the depth of participant responses about food access, though accessing food was a topic initiated by many of our study participants. However, accessing food was one of the most important utilitarian activities that our participants needed to accomplish, and food locations were the most common destination visited while participants wore the GPS device. This finding must be cautiously interpreted given that 3 days is a relatively short time period to monitor mobility, but it illustrates the importance of addressing food access among this population of midlife and older adults with mobility disabilities. Other limitations of the study include that we specifically recruited participants who leave their homes at least 3 days per week, so we did not obtain the perspectives of those who are home-bound and likely face additional challenges to accessing food. Additionally, our participants were fairly culturally homogeneous (predominantly Caucasian and female).

The key strengths of our study are that it provides insightful information about where midlife and older adults with mobility disability accessed food, how they travelled to these destinations, reasons for accessing particular food destinations, and their perceptions of the indoor built environment and other factors at these destinations. Our use of qualitative methods allowed us to obtain information that would be otherwise difficult to capture using other research methods. Our findings are difficult to compare to previous studies, because these studies have not examined the types of destinations older adults with disabilities access for food, or many of the facilitators or barriers to food access. Since there is a lack of previous studies available about this topic, we feel our findings provide useful information.

Accessing food is essential to life and can become more difficult with older age and mobility disability. Consideration needs to be given to how our aging population will access food, principally where grocery stores are located and proximity to different modes of transportation (especially public transit where available). Urban planning should particularly consider proximity of food access destinations to other destinations with goods and services, as this may help older adults continue to live independently in the community. Attention to easy pedestrian access (e.g., short, well-marked, and protected routes) would facilitate visiting multiple destinations. Food access destinations should also take into account the needs of this population, especially the need for adequate space and ease of entry while using an assistive device. Future studies should include determining how food access can be better understood and improved in order to prepare for the growing population of adults aging with a mobility disability. Improved understanding of how the types of food destinations relate to caloric intake and diet quality among older adults with mobility disabilities would also be helpful for future research efforts. Overall, further research of maintaining food access for adults aging with mobility disability would ideally impact public policy, urban planning, and businesses to help older adults function and thrive in their communities.

## Figures and Tables

**Table 1:** Characteristics of study participants (*n* = 35).

Characteristic	Value
Age in years, mean ± SD (range)	66.8 ± 9.4 (50–86)
Sex, *n* (%)	
Male	9 (25.7)
Female	26 (74.3)
Race/ethnicity, *n* (%)	
Caucasian	30 (85.7)
African-American	2 (5.7)
Asian	1 (2.9)
≥1 race	1 (2.9)
Walkscore, mean (range)	67 (18–98)
Household income, median (range)	$46,199 ($25,821–$94,179)
Reside in low-income (<$35,000) census tract, *n* (%)	9 (25.7)
Reside in food desert, *n* (%)	2 (5.7)
Participants living in facilities that provide meals, *n* (%)	4 (11.4)
Type of assistive device, *n* (%)	
Cane	20 (57.1)
Walker (2- or 4-wheeled)	20 (57.1)
Electric wheelchair or scooter	9 (25.7)
Manual wheelchair	7 (20)
Other	4 (11.4)
Participants who drive, *n* (%)	7 (20)

**Table 2:** Factors impacting choice of food access destinations.

Factors	Facilitators	Barriers
	Theme/concept	Theme/concept
Location	Close proximity/ease of travel	Long distance
	Proximity to other locations (e.g., other goods and services) to group errands	
	Lower cost/affordability	Higher cost
	Facility follows ADA Standards for Accessible Design	Difficult entry
	Larger size of location	Small size of location
		Lack of handicapped parking
		Crowded parking lot
	Product availability and selection	

Participant	Preference for store and products	Limited by product selection
	Smaller shopping trips	Difficulty carrying purchases
	Leave home to eat, socialize, activity	

Outdoor built environment		Lack of sidewalks
		Obstructed roads
		Highways
		Hills
		Lack of public transit

**Table 3:** Facilitators and barriers of food access destinations.

Theme	Facilitators	Barriers
	Concept/theme	Concept/theme
Space	Adequate space	Inadequate space
	Ease of navigation	Navigation difficulty
	Helpful general features	Unhelpful general features

Entry/accessibility	Ease of entering destination	Difficulty entering destination

Amenities	Seating available	Seating unavailable
	Restrooms	Restroom features inadequate
	Drinking fountains	
	Shopping cart as assistive device for mobility	
	Availability of electric shopping carts	Lack of electric shopping carts

People	Good service	Poor/suboptimal service
	Helpful family, friends, and caregivers	Inattentive fellow customers
